# Longxuetongluo Capsule Improves Erythrocyte Function against Lipid Peroxidation and Abnormal Hemorheological Parameters in High Fat Diet-Induced ApoE**−/−** Mice

**DOI:** 10.1155/2016/2603219

**Published:** 2015-11-16

**Authors:** Jiao Zheng, Binglin Liu, Qixing Lun, Weijuan Yao, Yunfang Zhao, Wei Xiao, Wenzhe Huang, Yonghua Wang, Jun Li, Pengfei Tu

**Affiliations:** ^1^Modern Research Center for Traditional Chinese Medicine, Beijing University of Chinese Medicine, Beijing 100029, China; ^2^School of Chinese Materia Medica, Beijing University of Chinese Medicine, Beijing 100102, China; ^3^Hemorheology Center, School of Basic Medical Sciences, Peking University Health Science Center, Beijing 100191, China; ^4^National Key Laboratory of Pharmaceutical New Technology for Chinese Medicine, Jiangsu Kanion Pharmaceutical Co. Ltd., Lianyungang 222001, China

## Abstract

Chinese dragon's blood, the red resin of *Dracaena cochinchinensis*, one of the renowned traditional medicines, has been used to facilitate blood circulation and disperse blood stasis for thousands of years. Phenolic compounds are considered to be responsible for its main biological activities. In this study, total phenolic compounds of Chinese dragon's blood were made into capsule (Longxuetongluo Capsule, LTC) and their effects on the abnormal hemorheological properties were examined by high fat diet (HFD) induced ApoE−/− mice. Compared to the model group, LTC recovered the abnormal hemorheological parameters in HFD-induced ApoE−/− mice by reducing whole blood viscosity (WBV) at high rate and improving erythrocyte function. In conclusion, LTC could ameliorate erythrocyte deformability and osmotic fragility through the reduction of lipid peroxidation on plasma and erythrocyte membranes in HFD-induced ApoE−/− mice, which supported the traditional uses of Chinese dragon's blood as an effective agent for improving blood microcirculation in hypercholesterolemia.

## 1. Introduction

Hyperlipidemia, a metabolic derangement, is a condition characterized by high levels of cholesterol in the blood, which is an important risk factor for developing atherosclerosis and cardiovascular diseases. Numerous clinical trials have focused on the effect of atherogenic lipids on hemorheological factors associated with cardiovascular diseases, such as increased fibrinogen [1], erythrocyte aggregation [2], whole blood viscosity (WBV) [3], plasma viscosity [4], decreased osmotic fragility and deformability [3], and decreased plasma membrane fluidity [5]. Among them, the function of erythrocyte is one of the most important factors in the changes of hemorheological parameters. Cholesterol is an essential and functional component of cell membranes, and any change in cholesterol levels of erythrocyte membrane reflects substantial modification of the serum lipid profile since no* de novo* cholesterol synthesis occurs in the erythrocytes [6]. Cholesterol enrichment in erythrocytes may cause impairment of their functional properties including rheological behaviors, such as osmotic fragility and deformability, which can result in atherosclerotic lesions [7]. Peroxidation of membrane lipids can result in the inactivation of enzymes and cross-linking of membrane lipids and proteins. Many lines of evidences suggest that oxidative erythrocyte contributes directly to the pathogenesis of cardiovascular diseases due to cell death and inflammation [8, 9].

It has been reported that the medical treatment of hyperlipidemia had positive effects on the abnormal hemorheological parameters in previous literature [10]. Ezetimibe is the first lipid-lowering drug that inhibits intestinal uptake of dietary and biliary cholesterol without affecting the absorption of fat-soluble nutrients. The standard dose of 10 mg/day lowers low density lipoprotein cholesterol (LDL-c) by 15–20% when used alone. Therefore, the ezetimibe had been used as a positive control agent for lipid-lowering effect in this study.

Chinese dragon's blood is the red resin of* Dracaena cochinchinensis* (Lour.) S. C. Chen and has been used as a famous traditional medicine for centuries. It has been used to stimulate circulation, promote tissue regeneration by aiding the healing of fractures, sprains, and ulcers, and control bleeding and pain [11]. Previous phytochemical investigations have revealed that Chinese dragon's blood is rich in flavans, flavonoids, isoflavonoids, chalcones, sterols, and terpenoids, and its main biological activities come from its phenolic compounds [12]. The pure compounds and crud extracts from Chinese dragon's blood have been reported to possess a wide array of pharmacological activities, such as anti-inflammatory activity [13], thrombin inhibitory effects [14], antithrombotic properties [15], and antiplatelet aggregation [16]. In June 2013, Longxuetongluo Capsule (LTC), a new drug consisting of the total phenolic extract of Chinese dragon's blood, was approved for the treatment of ischemic stroke by China Food and Drug Administration after phases II (116 cases) and III (348 cases) of clinical trials.

As for the antioxidant property of LTC, some studies suggested that it could reduce the injuries due to the radiation-induced oxidative stress both* in vitro* and* in vivo* [17, 18]. However, little is known about the effects of LTC on abnormal hemorheological parameters and its antioxidative activity on erythrocyte membrane in hyperlipidemia. The aim of this study was to investigate the effects of LTC on erythrocytes function in ApoE−/− mice suffering from hypercholesterolemia that was induced by high fat diet (HFD).

## 2. Materials and Methods

### 2.1. Animals

Male ApoE−/− mice, weighing 19–23 g, were obtained from the Animal Center of Peking University Health Science Center. All the experiments were approved by the local Medical Ethics Committee. The mice were maintained at 24 ± 1°C and a relative humidity of 50 ± 1% with a light/dark cycle of 12 h. After one week of feeding up with the HFD (0.2% cholesterol and 15% fat added), the mice were randomly divided into five groups (*n* = 10 for each group): the HFD-induced group (HG) and the low, middle, and high doses of LTC and ezetimibe (Schering-Plough Pte Ltd., lot:2EZPA17005) treatment groups (LTC100 group, LTC200 group, LTC300 group, and EG, resp.), and they were administered orally with 100, 200, and 300 mg/kg LTC and 30 mg/kg ezetimibe once a day, respectively. For each group, the food and water were available* ad libitum* for successive six weeks.

### 2.2. Medicinal Materials

**Figure 1:**
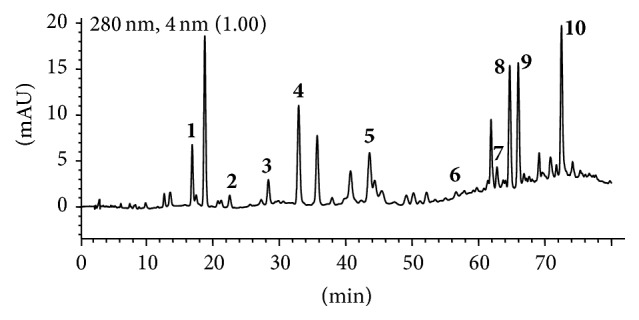
High-performance liquid chromatogram of LTC at 280 nm.** 1**: 7,4′-dihydroxyflavone;** 2**: loureirin D;** 3**: 7,4′-dihydroxyhomoisoflavanone;** 4**: loureirin C;** 5**: 3,4′-dihydroxy-5-methoxystilbene;** 6**: 5,7-dihydroxy-4′-methoxy-8-methylflavan;** 7**: 4-hydroxy-2,4′-dimethoxydihydrochalcone;** 8**: loureirin A;** 9**: loureirin B; and** 10**: pterostilbene.

**Figure 2:**
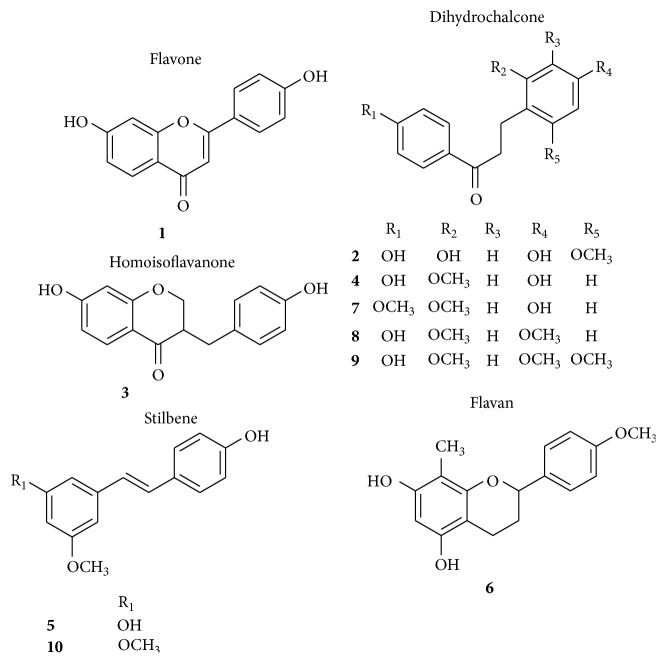
Chemical structures of the main components in LTC.

LTC was provided by Jiangsu Kanion Pharmaceutical Co. Ltd. (Jiangsu, China) [19–21], and the content of total phenols accounted for 70.07% as determined by colorimetry method [21]. The chemical profile of LTC is shown in Figure 1. HPLC analysis was performed according to the previous method with slight modification [20, 21]. Briefly, the sample was run on an Agilent XDB-C_18_ column (250 × 4.6 mm i.d., 5 *μ*m, Agilent Technologies, Palo Alto, CA, USA) and the column temperature was set at 30°C. The mobile phase was composed of acetonitrile (A) and 0.1% aqueous formic acid (B) and delivered at a total flow rate of 1.0 mL/min following a gradient program: 0–35 min, 20%–31% A; 35–45 min, 31%–31% A; and 45–80 min, 31%–55% A; UV absorption over 190–400 nm was recorded by Diode Array Detector (DAD), and fixed wavelength of 280 nm was used for the detection of phenolic constituents. The injection volume was set at 10 *μ*L. Ten phenolic compounds in LTC were identified as 7,4′-dihydroxyflavone (**1**, *t*_*R*_ 16.91 min), loureirin D (**2**, *t*_*R*_ 22.56 min), 7,4′-dihydroxyhomoisoflavanone (**3**, *t*_*R*_ 28.39 min), loureirin C (**4**, *t*_*R*_ 32.93 min), 3,4′-dihydroxy-5-methoxystilbene (**5**, *t*_*R*_ 43.59 min), 5,7-dihydroxy-4′-methoxy-8-methylflavan (**6**, *t*_*R*_ 56.61 min), 4-hydroxy-2,4′-dimethoxydihydrochalcone (**7**, *t*_*R*_ 62.78 min), loureirin A (**8**, *t*_*R*_ 64.70 min), loureirin B (**9**, *t*_*R*_ 65.98 min), and pterostilbene (**10**, *t*_*R*_ 72.48 min) by comparison of their retention times and UV spectra with those of authentic compounds [22, 23]. The structures of the identified compounds are shown in Figure 2. Furthermore, the content of two main phenolic compounds, 7,4′-dihydroxyflavone (0.69%) and loureirin B (0.83%), in the total phenol extract of LTC was determined by using our patented analytical method [21].

### 2.3. Blood Collection and Measurements of Plasma Lipids and Malondialdehyde (MDA)

The blood for the following hemorheological measurements was collected from the mice which were fasted for 6 hours. Blood was anticoagulated by heparin for the following measurements. Levels of total cholesterol (TC), triglycerides (TG), and low density lipoprotein cholesterol (LDL-c) in plasma were analyzed enzymatically using commercial kits (Biosino Bio-technology and Science Inc., Beijing, China). MDA levels of plasma were measured by a commercial kit (Nanjing Jiancheng Bioengineering Institute, Nanjiang, China).

### 2.4. Measurements of WBV and Hematocrit (Hct)

The WBV was measured at shear rates of 200, 50, and 1 s^−1^ in a capillary viscometer (Model LG-R-80B, Steellex Co., China) at 37°C. After the heparin anticoagulated blood in a capillary tube was centrifuged (1000 g, 10 min), Hct was determined through measuring the length of column of the erythrocytes and the whole blood, respectively [24]. Erythrocyte aggregation index (AI) was calculated according to the equation written as AI = *η*_*L*_/*η*_*H*_, in which *η*_*L*_ was for the value of WBV at low shear rate of 1 s^−1^ and *η*_*H*_ was for the value of WBV at relatively high shear rate of 200 s^−1^ [25].

### 2.5. Erythrocyte Deformability, Osmotic Fragility, and Scanning Electron Microscopy

The measurement of erythrocyte deformability was carried out at 37°C using an ektacytometer (Model LBY-BX2, Precil Co., China). Erythrocyte deformation index (DI) was calculated by shear rates ranging from 50 to 1000 s^−1^ as DI = (*L* − *W*)/(*L* + *W*), in which *L* and *W* are for the length and width of the elliptical diffraction pattern, respectively [26]. The osmotic fragility of erythrocytes was measured according to previous literature [27]. The values of (DI)_max_ and deformation curve integral area were recorded. 20 *μ*L of blood was mixed with 2 mL of each dilution phosphate buffer solution (PBS) and incubated for 20 min at room temperature. After gentle centrifugation, the absorbance of the supernatant was determined at 540 nm. The data of *A* 540 nm of each sample in water was taken as 100% lysis, and readings of the same sample in various osmolarities solutions were normalized. For morphological analysis, fresh erythrocyte were fixed with 2% glutaraldehyde in PBS (pH 7.0) for 1 h and dehydrated with an ascending ethanol series (50–100%). The samples were dried and then coated with gold in an ion-coater apparatus. A scanning electron microscope (FEI, Quanta 250, USA) with accelerating voltage of 15 kV was used for observation [28].

### 2.6. SDS-PAGE and MDA Analysis of Erythrocyte Membranes

Erythrocytes were separated from blood plasma and leukocytes by means of centrifugation (3,000 g, 10 min) at 4°C and the plasma and the buffy coat were removed by aspiration [29]. Packed cells were washed in PBS three times successively. Then the erythrocytes were suspended in a cold 5 mM sodium phosphate buffer (pH 7.8) and incubated at 4°C for 10 min. The pellet was washed with PBS and centrifuged at 12,000 g for 10 min at 4°C for another three times. The “white” ghosts were estimated by the BCA method (Applygen Technologies Inc., Beijing, China) using bovine serum albumin as the standard and then frozen at −80°C until use [30]. Erythrocyte ghost proteins were separated on 4–12% polyacrylamide gels and stained with Coomassie Blue. MDA levels of erythrocyte ghosts were measured by a commercial kit (Nanjing Jiancheng Bioengineering Institute, Nanjiang, China).

### 2.7. Analysis of Erythrocyte Membrane Lipids

Erythrocytes were washed three times with 3 volumes of normal saline by centrifugation at 1,000 g for 5 min and subsequently removed the supernatant and the remaining buffy coat. The resulting erythrocyte pellet was suspended in ice-cold 20 mM phosphate buffer (pH 7.4), and centrifugation was carried out at 20,000 g for 20 min at 4°C. Washes in 20 mM phosphate were repeated until the erythrocyte ghost pellet was white. The erythrocyte membrane protein content was estimated by the BCA method (Applygen Technologies Inc., Beijing, China) using bovine serum albumin as standard. Erythrocyte membrane lipids were extracted by a dichloromethane-methanol solvent system at a ratio of 2 : 1 (v/v), dried under nitrogen, and finally solubilized in 5% Triton X-100. Aliquots of lipid extracts were taken for quantifying free cholesterol (FC) with a commercial kit (Applygen Technologies Inc., Beijing, China).

### 2.8. Statistical Analysis

Data were presented as means ± SEM. Statistical significance was determined by analysis of one-way ANOVA followed by Dunnett's post hoc test or Student's *t*-test for unpaired observations if appropriate. *P* < 0.05 was considered to be significant.

## 3. Results

### 3.1. Changes in Blood TC, TG, LDL-c, and MDA in the HFD-Induced Mice

**Figure 3:**
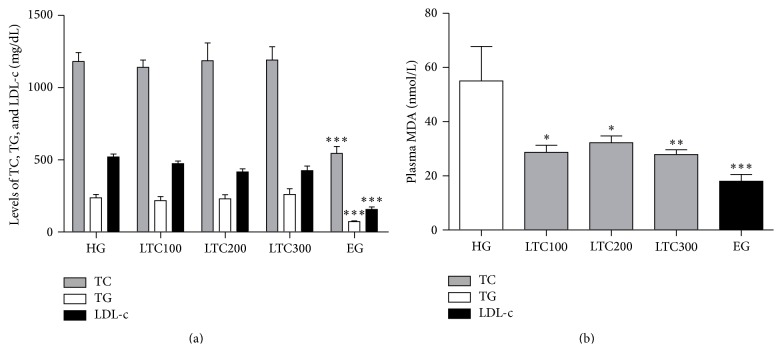
TC, TG, and LDL-c levels and MDA levels in plasma of ApoE−/− mice obtained after six weeks of drugs treatment. (a) TC, TG, and LDL-c levels in plasma. (b) MDA levels in plasma. The results reflect the outcome of experiments conducted on 9~10 mice for each measurement. ^*∗*^ *P* < 0.05 versus HG; ^*∗∗*^ *P* < 0.01 versus HG; ^*∗∗∗*^ *P* < 0.001 versus HG. EG: ezetimibe-treated group, HFD: high fat diet, HG: HFD-induced group, LDL-c: low density lipoprotein cholesterol, LTC: Longxuetongluo Capsule, LTC100 group: low dose LTC-treated group, LTC200 group: middle dose LTC-treated group, LTC300 group: high dose LTC-treated group, MDA: Malondialdehyde, TC: total cholesterol, and TG: triglycerides.

Before drug treatment, the ApoE−/− mice were fed with the HFD for one week, which significantly increased the TC, TG, and LDL-c levels by approximately 3 times, respectively (data not shown). Ezetimibe (trademark name Zetia) is a potent cholesterol absorption inhibitor that is being clinically used to treat hypercholesterolemia [31]. Ezetimibe was used as a positive contrast drug for the following study. After six weeks' treatment, ezetimibe reduced TC levels from 1180.8 to 544.379 mg/dL (46.10%; *P* < 0.001), TG levels from 237.364 mg/dL to 71.3628 mg/dL (30.06%; *P* < 0.001), and LDL-c levels from 512.8 mg/dL to 193.1 mg/dL (37.66%; *P* < 0.001) compared to the untreated mice fed with the same HFD (Figure 3(a)). However, all doses of LTC did not affect the TC, TG, HDL, and LDL-c levels in ApoE−/− mice. Lipid peroxidation, which refers to the oxidative degradation of lipids resulting in cell membrane damage, is considered a predictive biomarker for atherosclerosis and cardiovascular diseases [32]. Then the end products of lipid peroxidation (MDA) are tested in our following research. The data showed that the lower plasma MDA concentrations were observed after all drugs treatment (*P* < 0.05, Figure 3(b)). Compared with the TLC treatments, the ezetimibe-treated mice had the better MDA-lowering effect. The antilipid peroxidation activity of ezetimibe has been reported in previous literatures [33].

### 3.2. Changes in Blood WBV, Hct, and AI in the HFD-Induced Mice

In the current study, the cholesterol-lowering treatment (ezetimibe) reduced WBV in all the shears of ApoE−/− mice fed with HFD (*P* < 0.05, Table 1). However, all doses of LTC only significantly decreased WBV at 200 s^−1^. Erythrocyte counts and aggregated index (Hct and AI measurements) did not show significant differences between the groups, indicating that LTC had no influence on the number and aggregation of erythrocytes (Table 1).

### 3.3. LTC Improved the Erythrocyte Deformability and Osmotic Fragility

**Figure 4:**
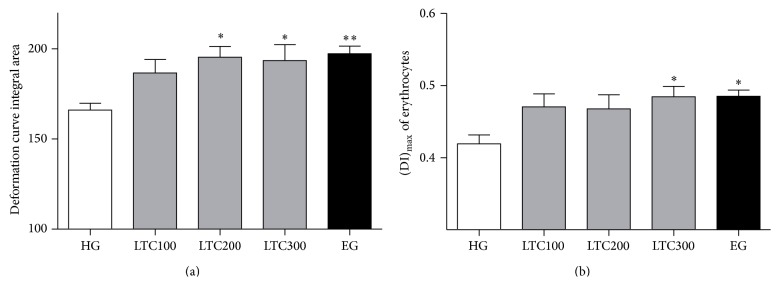
Deformability of erythrocytes in ApoE−/− mice. (a) The deformation curve integral area of erythrocytes. (b) The (DI)_max_ of erythrocytes. The results reflect the outcome of experiments conducted on 9~10 mice for each measurement. ^*∗*^ *P* < 0.05 versus HG; ^*∗∗*^ *P* < 0.01 versus HG. DI: erythrocyte deformation index, HFD: high fat diet, HG: HFD-induced group, LTC: Longxuetongluo Capsule, LTC100 group: low dose LTC-treated group, LTC200 group: middle dose LTC-treated group, and LTC300 group: high dose LTC-treated group.

Because erythrocyte deformability is a major factor of WBV at higher shear rates [34], LTC could improve the erythrocyte deformability in mice suffering from hypercholesterolemia. The changes of erythrocyte deformability and osmotic fragility had been investigated in the following studies. The erythrocyte deformability and osmotic fragility reflect the combination of membrane flexibility [35]. Previous studies on human subjects suggested that the erythrocyte deformability index (DI)_max_ significantly decreased in hypercholesterolemia [36]. Our study found that the deformation curve integral area of erythrocyte increased after treatment of ezetimibe and LTC (*P* < 0.05), and the significant difference occurred at doses of 200 and 300 mg/kg of LTC-treated groups (*P* < 0.05, Figure 4(a)). Meanwhile, the (DI)_max_ of erythrocyte significantly raised after 300 mg/kg of LTC or ezetimibe treatment (*P* < 0.05, Figure 4(b)).

**Figure 5:**
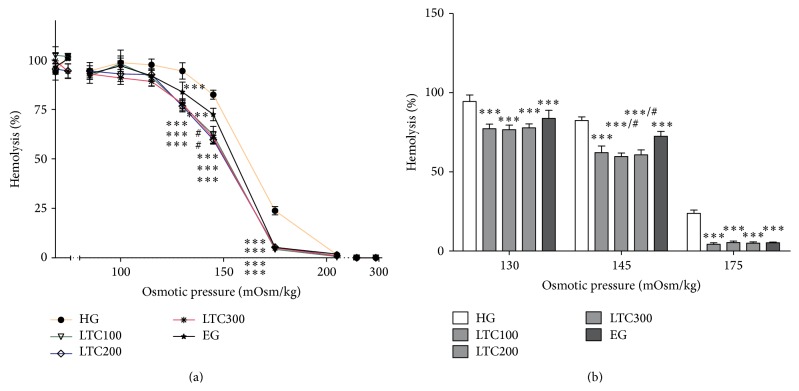
Osmotic fragility of erythrocytes in ApoE−/− mice of HG, LTC groups (100, 200, and 300 mg/kg), and EG: the percentages of hemolysis (%) rate against osmotic pressures (mOsm/kg). The results reflect the outcome of experiments conducted on 9 ~ 10 mice for each measurement. ^*∗∗∗*^ *P* < 0.001 versus HG; ^#^ *P* < 0.05 versus EG. HFD: high fat diet, HG: HFD-induced group, LTC: Longxuetongluo Capsule, LTC100 group: low dose LTC-treated group, LTC200 group: middle dose LTC-treated group, and LTC300 group: high dose LTC-treated group.

The osmotic fragility of erythrocytes reflects their membrane ability to maintain the structural integrity. As shown in Figure 5(a), all the erythrocytes remained intact at 0.01 mol/L PBS solution (295 mOsm/kg) in each group. The percentage of fragmentation rose gradually with the decrease of PBS concentration. From the osmotic pressure 130 to 175 mOsm/kg, integrated erythrocytes appeared more frequently in all the doses of LTC- and ezetimibe-treated mice than in those of untreated ones, which caused the left-shift of osmotic fragility curve, indicating that the erythrocytes of drug-treated mice were healthier compared to those of the HFD-induced mice. Besides, at the osmotic pressure of 145 mOsm/kg, the antihemolysis effects of TC200 and LTC300 treatments were better than that of ezetimibe treatment (*P* < 0.05) (Figure 5(b)), indicating that LTC was more effective in the improvement of osmotic fragility than ezetimibe.

**Figure 6:**
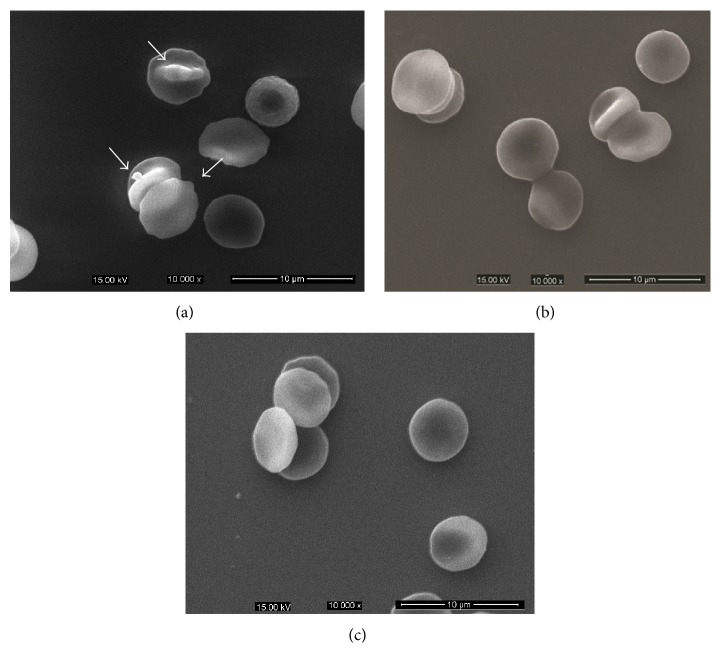
Morphological changes of erythrocytes in HFD-induced and LTC-treated mice observed with scanning electron microscope. (a) Erythrocytes of HFD-induced mice deformed with protrusions or irregular appearances. (b) Erythrocytes of LTC-treated (300 mg/kg) mice presented the more typical discocytes with rare deformed cells. (c) Erythrocytes of ezetimibe-treated mice presented the more typical discocytes with rare deformed cells.

The scanning electron microscopy demonstrated profound morphology alterations of erythrocytes by LTC treatment at dose of 300 mg/kg as shown in Figure 6. For the HFD-induced mice, most erythrocytes were deformed with protrusions or irregular appearances (Figure 6, arrows), and the abnormal erythrocyte morphology had been improved after LTC treatment.

### 3.4. LTC Inhibited the Lipid Peroxidation of Erythrocyte

**Figure 7:**
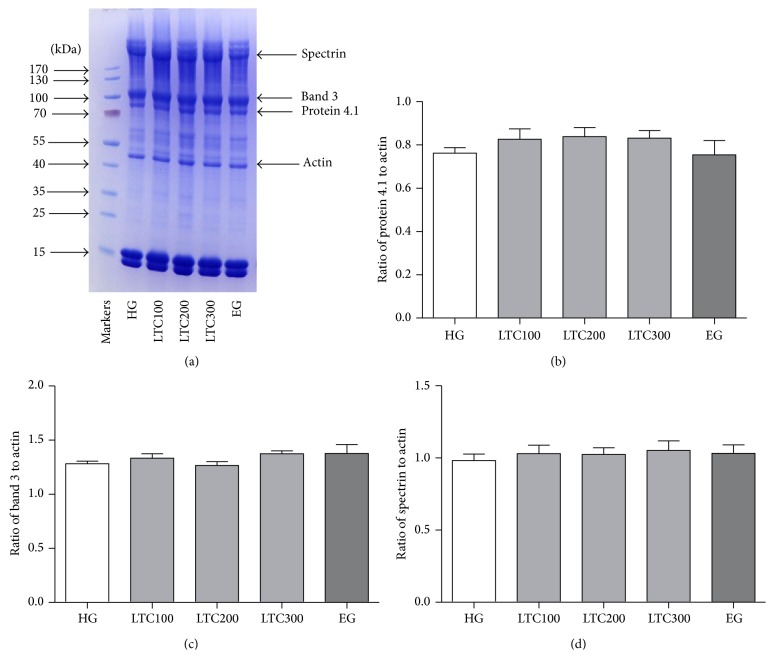
Effects of LTC on the association of erythrocyte skeleton. (a) The erythrocyte membrane ghosts were separated by SDS-PAGE. Here was the identification of the major erythrocyte membrane proteins. ((b), (c), and (d)) The quantitative results of the ratio of protein 4.1, band 3, and spectrin to actin. The results reflect the outcome of experiments conducted on 6 ~ 10 mice for each measurement. HFD: high fat diet, HG: HFD-induced group, LTC: Longxuetongluo Capsule, LTC100 group: low dose LTC-treated group, LTC200 group: middle dose LTC-treated group, and LTC300 group: high dose LTC-treated group.

**Figure 8:**
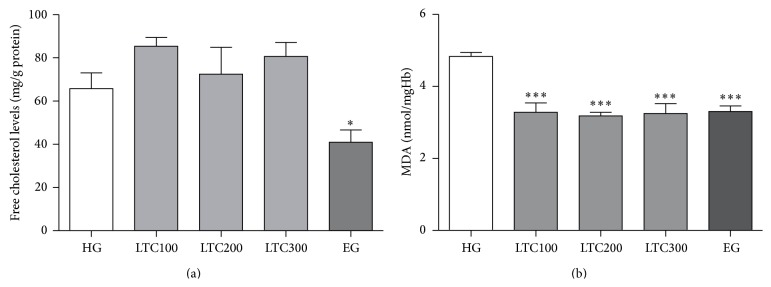
Effects of LTC on the lipid content and lipid peroxidation of erythrocyte membrane. (a) FC of erythrocyte membranes among HG, LTC groups (100, 200, and 300 mg/kg), and EG. (b) Effects of LTC on lipid peroxidation in erythrocyte membranes. ^*∗*^ *P* < 0.05 versus HG; ^*∗∗∗*^ *P* < 0.001 versus HG. FC: free cholesterol, HFD: high fat diet, HG: HFD-induced group, LTC: Longxuetongluo Capsule, LTC100 group: low dose LTC-treated group, LTC200 group: middle dose LTC-treated group, LTC300 group: high dose LTC-treated group, MDA: Malondialdehyde.

Many factors are probably associated with the improved osmotic fragility and deformability in erythrocytes, such as the erythrocyte skeleton, membrane lipid composition, and lipid peroxidation. In this study, the erythrocyte skeleton was studied by SDS-PAGE of erythrocyte ghosts. No significant changes of the major proteins, such as *α*-spectrin (band 1), *β*-spectrin (band 2), band 3, and 4.1 in membrane protein fractions, were observed among LTC- and ezetimibe-treated and untreated mice (Figure 7). As for lipid composition of the erythrocyte membrane, only ezetimibe had significantly decreased the FC level of erythrocyte ghost (*P* < 0.01, Figure 8(a)).

Previous studies showed that the erythrocyte membrane was particularly sensitive to oxidative damage due to its high polyunsaturated fatty acid content [37]. Exposure of erythrocyte to hyperlipidemia leads to increased concentration of lipid peroxidation [38]. The lower MDA levels in erythrocytes were obviously observed after drugs treatment (Figure 8(b)). These results suggested that LTC improved erythrocyte deformability and osmotic fragility partly may at least due to its antilipid peroxidation effect.

## 4. Discussion

As one of the major indicators of hemorheology, WBV can be described as the thickness and stickiness of blood [39]. The primary determinants of WBV are Hct, erythrocyte deformability and aggregation, and plasma viscosity [40]. Previous studies on human subjects showed that hypercholesterolemia resulted in increased viscosity of plasma and blood [41]. The contribution of hypercholesterolemia to viscosity was dependent on the concentration of the cholesterol-rich lipoproteins [39]. Others had found that addition of isolated LDL-c caused a dose-dependent and exponential rise in plasma viscosity [4]. In another study, cholesterol levels had been shown to be significantly correlated to plasma viscosity after cholesterol-lowering treatment. Ezetimibe, a novel cholesterol-lowering drug, could significantly decrease the TC, TG, and LDL-c levels in plasma, but LTC had no effect on lipids levels in the plasma of ApoE−/− mice. Since the TC, TG, and LDL-c in plasma of LTC-treated mice remained with no significant change compared with those of HG treatment, we could deduce that LTC did not influence the plasma viscosity. Erythrocyte deformability is a major factor for the WBV at higher shear rates, while the changes of lower shear rate came from erythrocyte aggregation. In the present study, a sharp decrease of WBV was observed at 200 s^−1^ after LTC treatment, which indicated that LTC could improve the erythrocyte deformability.

Osmotic fragility, which is the indication of the integrity of the erythrocyte membranes, exhibits the ability of erythrocyte membranes to endure the hypotonic pressures. The mechanisms for altering osmotic fragility have been reported as follows: erythrocyte surface area/volume ratio, membrane cholesterol [42], removal of glycoproteins and band 3 [43], deficiencies in band 4.2 [44], insertion of fatty acids [45], action of phospholipases [45, 46], Na-K-2Cl cotransport activity [45], and antioxidant status of erythrocytes [47]. In the present study, the osmotic fragility of erythrocytes of all doses of LTC-treated mice had been markedly improved from 130 to 175 mOsm/kg, and the efficacy of 200 mg/kg and 300 mg/kg LTC treatment was better than that of ezetimibe treatment which had an excellent lipid-lowering activity.

To characterize the morphology of erythrocytes, we utilized scanning electron microscopy to visualize the differences in membrane topography caused by hyperlipidemia. Microcytic vesicles are seen budding off the erythrocytes of HG mice while less microcytic vesicles and protruding structures were observed in LTC and EG treated erythrocytes. In order to figure out the mechanism underlying the effect of LTC on improving the osmotic fragility of hypercholesterolemic mice, the skeleton, FC and oxidation levels of erythrocyte membrane were examined.

The erythrocyte skeleton is critical for the mechanical properties of this cell. It is formed by a complex meshwork of proteins which imparts a great degree of characteristic shape and elasticity [48]. Spectrin, actin, protein 4.1, and dematin were the abundant proteins in the erythrocyte membrane cytoskeleton and played a critical role in the maintenance of erythrocyte elasticity and membrane mechanical properties. Analysis of erythrocyte membrane proteins by gel electrophoresis showed that the ratios of spectrin, protein 4.1, and band 3 to actin remained essentially unaltered in the ghosts of LTC-treated mice, implying that the improvement of osmotic fragility was not due to the ratios of main proteins alternations of erythrocyte membranes.

Recent studies have indicated that the FC in erythrocyte membrane could structurally contribute to plaque formation as well as serum lipoprotein fractions [7]. Lipid composition of the erythrocyte membrane is the primary determinant of membrane fluidity, membrane deformability, and erythrocyte deformability. Lipids exchanged between erythrocyte membrane and plasma both* in vivo* and* in vitro* and lipids in erythrocyte membrane would increase due to the high levels of plasma lipids [49]. Similar to the previous report, the significant decrement of FC in erythrocyte membranes of ezetimibe-treated ApoE−/− mice was affected by its lipids-lowering effect, which indicated that the improvement of osmotic fragility in erythrocyte was associated with the decreased cholesterol content of membranes [29]. The lipid-lowering impact of ezetimibe was believed to result from an influence on cholesterol exchange by binding to the plasma membrane and displacing cholesterol between the lipoproteins and the erythrocytes [50]. Although LTC improved the osmotic fragility of erythrocyte, no changes of FC and membrane proteins were observed. This implied that there could be other mechanisms responsible for the improvement of LTC on the osmotic fragility of erythrocyte.

Clinical trials have shown that dyslipidemia contributes to the development of cardiovascular diseases and that this course is amplified by oxidative stress. Erythrocytes are very susceptible to the free radicals which cause structural and functional disorders in the cell membrane, thus decreasing the cell's capability of deformation [5]. As a measurement of oxidative stress [51], MDA levels in the erythrocyte and plasma of the drug-treated groups had been significantly decreased in both erythrocyte and plasma of subgroups compared to that of the HFD-induced mice. The remarkable decrease of MDA levels in both plasma and erythrocyte membranes indicated that LTC mitigated the production of lipid peroxidation.

## 5. Conclusions

Chinese dragon's blood has been used in traditional medicine to stimulate circulation and promote tissue regeneration for many years. It has been clinically used for the treatment of cerebral arterial thrombosis, ischemic heart disease, blood stasis syndrome, and so forth. Recently, LTC, a new drug consisting of the total phenolic extract of Chinese dragon's blood extract, has been used in clinical practices for the treatment of ischemic stroke. In the present study, we found that treatment of dyslipidemic mice with LTC not only caused favorable changes in blood rheology but also led to an antioxidative impact against oxidative stress, which ameliorated erythrocyte deformability and osmotic fragility and decreased the oxidized lipoproteins in plasma. The effects of LTC on blood rheology in hyperlipidemia suggested that LTC could be an effective agent to improve microcirculation and erythrocyte function.

## Figures and Tables

**Table 1:** LTC effects on WBV, Hct, and AI.

Groups	WBV (mPa·s) (*n* = 5)			Hct (%) (*n* = 6–8)	AI (*n* = 5)
	200 s^−1^	50 s^−1^	1 s^−1^		
HG	3.99 ± 0.15	5.92 ± 0.314	31.32 ± 1.58	53.11 ± 1.77	8.02 ± 0.56
LTC100	2.93 ± 0.22^*∗*^	4.56 ± 0.50	27.06 ± 2.89	52.91 ± 1.84	9.48 ± 0.85
LTC200	2.70 ± 0.15^*∗*^	4.95 ± 0.60	26.78 ± 1.74	50.39 ± 4.48	9.35 ± 1.00
LTC300	2.66 ± 0.19^*∗*^	4.72 ± 0.28	28.32 ± 1.87	48.78 ± 3.06	8.57 ± 0.68
EG	2.41 ± 0.18^*∗*^	3.84 ± 0.26^*∗*^	21.82 ± 1.19^*∗*^	55.41 ± 3.66	8.06 ± 0.31

Data are the mean ± SEM. ^*∗*^ *P* < 0.05 versus HG. AI: erythrocyte aggregation index, EG: ezetimibe-treated group, Hct: hematocrit, HFD: high fat diet, HG: HFD-induced group, LTC: Longxuetongluo Capsule, LTC100 group: low dose LTC-treated group, LTC200 group: middle dose LTC-treated group, LTC300 group: high dose LTC-treated group, and WBV: whole blood viscosity.

## Abbreviations

AI: Erythrocyte aggregation index
DI: Erythrocyte deformation index
DAD: Diode Array Detector
EG: Ezetimibe-treated group
FC: Free cholesterol
Hct: Hematocrit
HFD: High fat diet
HG: HFD-induced group
LDL-c: Low density lipoprotein cholesterol
LTC: Longxuetongluo Capsule
LTC100 group: Low dose LTC-treated group
LTC200 group: Middle dose LTC-treated group
LTC300 group: High dose LTC-treated group
MDA: Malondialdehyde
PBS: Phosphate buffer solution
TC: Total cholesterol
TG: Triglycerides
WBV: Whole blood viscosity.
